# Clinical characteristics and genetic features of patients with Wilson’s disease in southwestern China

**DOI:** 10.1186/s13023-026-04426-y

**Published:** 2026-06-03

**Authors:** Lu Zhang, Yao Dong, Jieru Peng, Denghui Yang, Bing Pang, Zhuo Tan, Qiwen Zhang, Zhong Li, Dailan Yang, Juan Liao, Chunxia Yang

**Affiliations:** 1 Department of Epidemiology and Biostatistics, West China School of Public Health and West China Fourth Hospital, Sichuan University, Chengdu, Sichuan 610041 China; 2 Department of Medical Records Statistics, Chengdu Women’s and Children’s Central Hospital, School of Medicine, University of Electronic Science and Technology of China, Chengdu, Sichuan 611731 China; 3 Department of Toxicosis/Nephrology, West China School of Public Health and West China Fourth Hospital, Sichuan University, Sichuan, 610041 China; 4 Department of Gastroenterology, West China School of Public Health and West China Fourth Hospital, Sichuan University, Chengdu, Sichuan 610041 China; 5 Non-communicable Diseases Research Center, West China-PUMC C.C. Chen Institute of Health, Sichuan University, Chengdu, Sichuan 610041 China

**Keywords:** Wilson’s disease, Clinical characteristics, ATP7B, Mutations, Genotype-phenotype correlation

## Abstract

**Background:**

Wilson’s disease (WD) is a rare autosomal recessive disorder caused by mutations in the ATP7B gene, leading to copper metabolism dysfunction. The clinical and genetic manifestations of WD vary across populations. This study aimed to characterize the clinical features and genetic mutations in WD patients from southwestern China and to explore genotype-phenotype correlations.

**Methods:**

A total of 120 WD patients from southwestern China were included. Clinical data were collected through face-to-face interviews and medical record reviews. ATP7B mutations were identified through whole-genome resequencing. Genotype-phenotype correlations were analyzed in patients with pathogenic mutations on both alleles.

**Results:**

Among the 120 WD patients, the median age at onset was 17 years (range 3 to 49 years), with neurological symptoms being the most common initial presentation (61.7%). At the time of enrollment, the median disease duration was 10 years, with persistent neurological symptoms commonly observed, and 62.5% of patients had progressed to cirrhosis. Sex and age differences in clinical characteristics were also observed. In the analysis of ATP7B gene mutations, a total of 47 distinct pathogenic mutations were identified, including 32 missense, 6 nonsense, 6 frameshift, and 3 splicing mutations. The most prevalent mutations were c.2333G > T (p.R778L) and c.2975 C > T (p.P992L). Notably, one novel pathogenic frameshift mutation, c.386delA (p.K129Rfs*24), and another novel variant of uncertain significance, c.1987 C > A (p.P663T), were identified. Among the 117 unrelated patients, 98 (83.8%) had pathogenic mutations on both alleles, with 62 different mutation combinations observed. Genotype-phenotype analysis showed that p.R778L, p.P992L, and loss-of-function (LOF) mutations were not significantly associated with the initial clinical subtype, whereas p.R778L was associated with a later age at onset and LOF mutations with an earlier age at onset.

**Conclusion:**

This study provided some valuable insights into the clinical and genetic characteristics of WD in southwestern China, enhanced the understanding of genotype-phenotype correlations, and emphasized the importance of early diagnosis and personalized management.

**Supplementary Information:**

The online version contains supplementary material available at 10.1186/s13023-026-04426-y.

## Introduction

Wilson’s disease (WD) is a rare autosomal recessive disorder of copper metabolism caused by pathogenic mutations in the ATP7B gene, which encodes a copper-transporting P-type ATPase [1]. Defective ATP7B function leads to excessive copper accumulation, primarily in the liver and brain, resulting in progressive hepatic and neurological damage. The global prevalence of WD is estimated to be between 1 in 30,000 and 1 in 50,000 [2]. However, recent studies suggest that the prevalence of WD may be underestimated [3–5].

WD manifests with hepatic, neurological, and psychiatric symptoms, which may appear individually or in combination, with variations across different age groups and sexes [1]. Hepatic involvement, often the earliest symptom, is more common in younger patients and may progress to cirrhosis and liver failure in advanced cases [6, 7]. Neurological symptoms typically emerge between the ages of 20 and 30, with some evidence suggesting an earlier onset and greater severity in males [8–10]. Psychiatric symptoms often occur with neurological manifestations but may also present as the initial clinical features [11, 12]. Additionally, excess copper deposition can contribute to renal, hematologic, cardiac, musculoskeletal, and endocrine abnormalities [13, 14].

ATP7B gene locates on the long arm of chromosome 13 (13q14.3), spanning approximately 85 kb and comprising 21 exons and 20 introns. It encodes an eight-transmembrane-domain protein composed of 1465 amino acids, which is localized to the trans-Golgi network (TGN) [15–17]. In hepatocytes, ATP7B facilitates the incorporation of copper into newly synthesized ceruloplasmin in the TGN and mediates copper excretion into bile when intracellular copper levels rise. Dysfunction of ATP7B function leads to the abnormal deposition of copper in the body. Excess copper triggers oxidative stress, mitochondrial dysfunction, disruption of gene regulation, and apoptosis [18]. Currently, over 1000 mutations, including substitutions, insertions, deletions, and duplications, have been identified in the ATP7B gene [19]. The distribution of ATP7B mutations differs among populations and geographic regions. The p.H1069Q (c.3202 A > C) and p.R778L (c.2333G > T) mutations, located in exons 14 and 8, respectively, are the most common pathogenic mutations in European and Asian populations [20].

WD patients exhibit considerable genetic and phenotypic heterogeneity. Previous studies showed that p.R778L is associated with an earlier age of onset, lower ceruloplasmin (Cp) levels, and higher zinc levels; p.R919G is linked to the neurological subtype, while p.A1295V is related to the hepatic subtype; Additionally, loss-of-function (LOF) mutations correlate with lower serum albumin levels [21–24]. However, the genotype-phenotype correlation in Chinese WD patients remains inconclusive, requiring further investigation and validation with data from different populations. This study aims to characterize the clinical and genetic features of WD patients in southwestern China, examine genotype-phenotype correlations, and expand the ATP7B mutation spectrum with genetic data from southwestern China.

## Methods

### Participants

Patients with WD who sought intravenous copper chelation therapy between August 2023 and July 2024 at West China School of Public Health and West China Fourth Hospital of Sichuan University were recruited. The clinical diagnosis of WD was based on a combination of clinical presentations and laboratory parameters, according to the professional guidelines [25]: age at onset (5–35 years old), symptoms (hepatic or neuropsychiatric), copper metabolism parameters (serum ceruloplasmin < 200 mg/L, 24-hour urinary copper excretion ≥ 100 µg, or liver copper > 250 µg/g dry weight), 24-hour urinary copper excretion > 1600 µg after following administration of 2 × 500 mg D-penicillamine, presence of Kayser-Fleischer rings observed via slit-lamp examination, positive family history, and genetic mutation analysis. Patients presenting with extrapyramidal or hepatic symptoms, positive K-F rings, serum ceruloplasmin < 200 mg/L, and 24-hour urinary copper excretion ≥ 100 µg can be diagnosed with WD without further examination. Additionally, the Leipzig score was referenced [26].

This study enrolled hospitalized patients with a confirmed diagnosis of WD. Exclusion criteria were as follows: (1) malignant tumors; (2) severe heart, pulmonary, haematological or toxic disease; (3) other hepatic diseases such as alcoholic, viral, autoimmune, drug-induced, or parasitic liver disease; (4) other neurological diseases such as Parkinson’s disease, primary tremor, or Huntington’s disease; (5) severe mental disorders; (6) important medical data missing; or (7) unwillingness to participate in this study. Each patient was recorded only once. Based on the inclusion and exclusion criteria, a total of 120 WD patients were included in the analysis.

This study was performed with the approval of the Ethics Committee of West China School of Public Health and West China Fourth Hospital (Gwll2023126) and adhered to the Declaration of Helsinki. All the written informed consent was obtained from participants. We confirm that all methods were executed according to the relevant guidelines.

### Clinical data collection

This study collected demographic and clinical data through face-to-face interviews and medical record reviews, including sex, age, family history of WD, disease duration, age at onset, initial symptoms, and clinical manifestations.

Patients were classified into four subtypes based on their initial symptoms, as described in previous literature [27]. Brefily, the hepatic subtype was primarily characterized by hepatic symptoms. The neurologic subtype included patients with neurological symptoms, with or without hepatic involvement. The asymptomatic subtype was identified incidentally during physical examinations. The other subtype included patients with other systemic symptoms.

### Mutation analysis of the ATP7B gene

Genomic DNA was extracted from the peripheral blood samples, and whole-genome resequencing was performed on 120 patients. All experimental protocols, including DNA extraction, library construction, high-throughput sequencing, and primary bioinformatic analysis, were conducted by Biomarker Technologies Co., Ltd (Beijing, China). The mean alignment rate was 99.93%, the mean sequencing depth was 31×, and the average genome coverage was 99.34% at ≥ 1×.

This study focused on mutations within the 21 exons of the ATP7B gene, and mutation annotation was performed based on the reference transcript NM_000053.4/ENST00000242839.10. Whether the mutations were known or novel was determined by referencing the gnomAD database (accessed on 14 November 2024, https://gnomad.broadinstitute.org/) and Human Gene Mutation Database professional (HGMD^®^ Professional 2024.3, accessed on 15 November 2024, http://www.hgmd.cf.ac.uk/).

The pathogenicity of the mutations were interpreted according to the standards and guidelines of the American College of Medical Genetics and Genomics and the Association for Molecular Pathology (ACMG/AMP) [28]. InterVar (http://wintervar.wglab.org/) was used to assist ACMG evidence annotation, and ClinVar (https://www.ncbi.nlm.nih.gov/clinvar/) together with the published literature served as important sources of previously reported evidence. The ACMG evidence codes applied to each pathogenic/likely pathogenic mutation are provided in Supplementary Table S1. Additionally, for missense mutations, computational prediction was performed using AlphaMissense score and VARITY_R score [29]. These results were used as supporting evidence for PP3/BP4 within the ACMG framework [29, 30].

For predicted LOF mutations, including nonsense, frameshift, and splice-site mutations, the applicability of PVS1 were evaluated according to the ClinGen recommendations [31], taking into account whether loss of ATP7B function is an established disease mechanism, the location of the mutation within the transcript, and the predicted likelihood of nonsense-mediated mRNA decay (NMD).

### Statistical analysis

R version 4.3.3 was used for data analysis. Results were presented as frequencies and proportions for categorical variables. All continuous numerical values were expressed as medians with ranges. Group comparisons for unordered categorical variables were conducted using the Chi-square test or Fisher’s exact test as appropriate. Comparisons of age at onset were analyzed using the Mann-Whitney test in the case of two groups, while inter-group comparisons for more than two groups were analyzed using the Kruskal-Wallis test. Associations of R778L, P992L, and LOF mutation carrier status with initial clinical subtypes were further examined using multinomial logistic regression, with asymptomatic onset as the reference category. Associations with age at onset were further assessed using linear regression. Sex was included as a covariate in adjusted analyses. *P* values were adjusted using the Benjamini-Hochberg false discovery rate (FDR) method where applicable, and adjusted *P* values were reported as *q* values. All tests were two-sided, and *P* or *q* < 0.05 was considered statistically significant, as appropriate.

## Results

### Clinical characteristics of WD patients

#### Basic information

Among 120 patients included in our study, 62 (51.7%) were female and 58 (48.3%) were male, with a median age of 30 years (range 12–57 years). A total of 32 patients (26.7%) had a positive family history of WD, with most of their affected relatives (90.6%) being siblings.

#### Initial symptoms

**Fig. 1:**
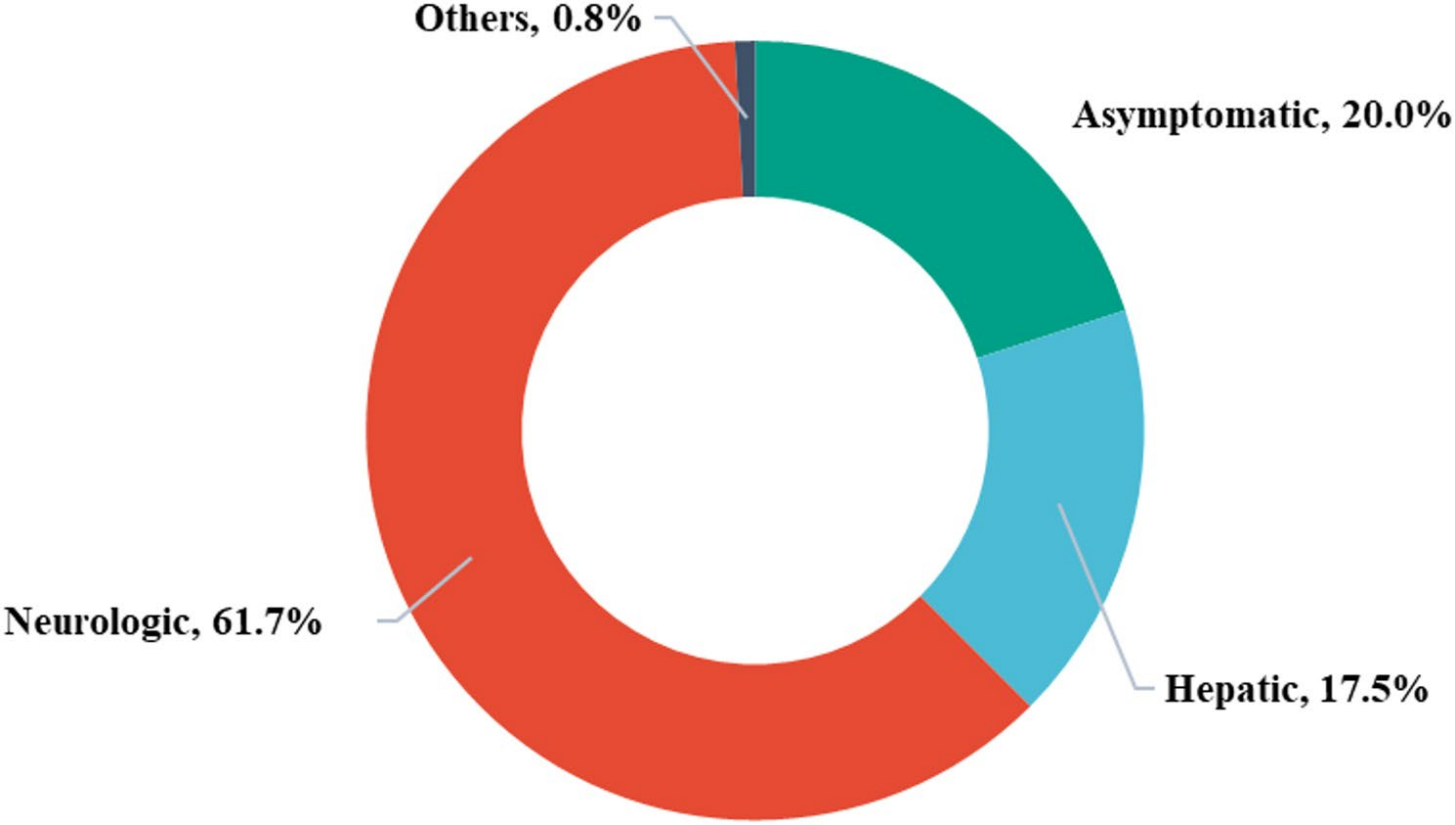
Initial clinical subtypes of WD patients

The median age at onset among the 120 patients was 17 years (range 3–49 years). Based on initial symptoms, most patients (61.7%) were classified as the neurologic subtype, 17.5% as the hepatic subtype, and 20.0% as the asymptomatic subtype. Only one patient, who presented with renal symptoms at onset, was classified as “Others” (Fig. 1). There were no significant differences in the distribution of initial symptom subtypes across onset age groups and between sexes.

Among the 120 patients with WD, neurological symptoms were the most common initial symptoms, including tremor (41.7%), dysarthria (22.5%), salivation (18.3%), gait abnormalities (15.0%), dystonia (9.2%), and swallowing difficulty (6.7%). 24 patients were asymptomatic but were identified during physical examinations, mainly by liver function tests suggesting elevation of transaminases (13.3%). Hepatic symptoms were less frequent, including jaundice (5.8%), lower limb edema (5.8%), abdominal distension (5.0%), abdominal pain (4.2%), diarrhea (4.2%), and nausea (4.2%). Only one patient started with other symptoms (nephritis).

As shown in Table 1, there were no significant differences in the distribution of predominant initial symptoms between males and females. However, the incidence of gait abnormalities varied significantly across different onset age groups (*P* = 0.006). Specifically, patients with an onset age of 21–25 years had a significantly higher proportion of gait abnormalities (35.7%) compared to those with an onset age of 15 years or younger (2.4%) (*P* < 0.05).

**Table 1:** Initial symptoms of patients with Wilson’s disease

Initial symptoms	Total (*n* = 120)	Sex			Age at onset (years)				
		Male **(*n*** **= 58)**	Female **(*n*** **= 62)**	*P* value	≤ 15 **(*n*** **= 42)**	16–20 ***(n*** **= 35)**	21–25 **(*n*** **= 14)**	> 25 **(*n*** **= 29)**	*P* value
**Subtypes**				0.965^*^					0.996^*^
Asymptomatic	24 (20.0)	12 (20.7)	12 (19.4)		8 (19.0)	8 (22.9)	3 (21.4)	5 (17.2)	
Hepatic	21 (17.5)	11 (19.0)	10 (16.1)		8 (19.0)	5 (14.3)	3 (21.4)	5 (17.2)	
Neurologic	74 (61.7)	35 (60.3)	39 (62.9)		25 (59.5)	22 (62.9)	8 (57.1)	19 (65.5)	
Others	1 (0.8)	0 (0.0)	1 (1.6)		1 (2.4)	0 (0.0)	0 (0.0)	0 (0.0)	
**Predominant initial symptoms**									
Tremor	50 (41.7)	20 (34.5)	30 (48.4)	0.123	15 (35.7)	15 (42.9)	7 (50.0)	13 (44.8)	0.765
Dysarthria	27 (22.5)	15 (25.9)	12 (19.4)	0.394	10 (23.8)	8 (22.9)	3 (21.4)	6 (20.7)	0.991
Salivation	22 (18.3)	14 (24.1)	8 (12.9)	0.112	11 (26.2)	8 (22.9)	1 (7.1)	2 (6.9)	0.116
Gait abnormalities	18 (15.0)	6 (10.3)	12 (19.4)	0.167	1 (2.4)	6 (17.1)	5 (35.7)^a^	6 (20.7)	**0.006** ^*^
Hepatic dysfunction	16 (13.3)	8 (13.8)	8 (12.9)	0.886	7 (16.7)	4 (11.4)	2 (14.3)	3 (10.3)	0.910^*^

Data are presented as *n* (%)

^a^ *P *< 0.05 when compared with ≤ 15 years of age at onset

^b^ *P* < 0.05 when compared with 16–20 years of age at onset

^c^ *P* < 0.05 when compared with 21–25 years of age at onset

^*^Fisher’s exact test

Bold values indicate statistically significant differences (*P* < 0.05)

#### Clinical manifestations

At the time of enrollment, the 120 patients had a median disease duration of 10 years (range 1–32 years). Among the 21 patients who initially presented with hepatic symptoms (hepatic subtype), 17 (81.0%) had progressed to cirrhosis, and 20 (95.2%) had developed neuropsychiatric symptoms. Of the 74 patients classified as the neurologic subtype at onset, the vast majority (98.6%) continued to experience neuropsychiatric symptoms, and 44 (59.5%) had developed cirrhosis. Among the 25 patients who were asymptomatic or had other initial presentations, 24 (96.0%) had varying degrees of liver damage currently, including 14 (56.0%) with cirrhosis, and 17 (68.0%) had developed neuropsychiatric symptoms.

Overall, 75 patients (62.5%) had cirrhosis, 59 (49.2%) had splenomegaly, and 52 patients (43.3%) felt fatigue at the time of enrollment. Neuropsychiatric symptoms were also prevalent, with over half of the patients displaying tremor (65.0%), dystonia (58.3%), dysarthria (57.5%), abnormal gait (52.5%), and salivation (51.7%). Additionally, 38 patients (31.7%) reported joint pain (Table 2).

By comparing the prevalence of predominant clinical symptoms across age groups and between sexes, we found that the prevalence of fatigue, memory impairment, and joint pain was higher in female patients (all *P* < 0.05), while male patients had a higher prevalence of portal hypertension (*P* = 0.001). Additionally, the prevalence of cirrhosis, jaundice, and sleep disorders varied significantly across different age groups (all *P* < 0.05). Among patients aged ≤ 20 years, the prevalence of cirrhosis was only 14.3%, whereas in the other three age groups, it exceeded 60%, reaching as high as 90.0% in those older than 40 years. Jaundice was also most prevalent in patients older than 40 years (45.0%). Similarly, sleep disorders were relatively uncommon in patients aged ≤ 20 years but were more frequently reported in the other three age groups, with prevalence rates of 31.7%, 44.7%, and 40.0%, respectively (Table 2).

**Table 2:** Clinical manifestations of patients with Wilson’s disease at the time of enrollment

Predominant clinical manifestations	Total (*n* = 120)	Sex			Age at enrollment (years)				
		Male **(*n*** **= 58)**	Female **(*n*** **= 62)**	*P* value	≤ 20 **(*n*** **= 21)**	21–30 ***(n*** **= 41)**	31–40 **(*n*** **= 38)**	> 40 **(*n*** **= 20)**	*P* value
Disease duration (years)	10 (6, 15)	9 (5, 14)	11 (6, 17)	0.176	6 (4, 10)	9 (6, 13)	14 (7, 18)^a^	12 (7, 21)^a^	**< 0.001**
**Hepatic**									
Cirrhosis	75 (62.5)	35 (60.3)	40 (64.5)	0.637	3 (14.3)	27 (65.9)^a^	27 (71.1)^a^	18 (90.0)^a^	**< 0.001**
Splenomegaly	59 (49.2)	26 (44.8)	33 (53.2)	0.358	8 (38.1)	17 (41.5)	24 (63.2)	10 (50.0)	0.173
Fatigue	52 (43.3)	19 (32.8)	33 (53.2)	**0.024**	7 (33.3)	22 (53.7)	16 (42.1)	7 (35.0)	0.358
Pruritus	39 (32.5)	19 (32.8)	20 (32.3)	0.953	6 (28.6)	13 (31.7)	11 (28.9)	9 (45.0)	0.614
Liver nodules	28 (23.3)	11 (19.0)	17 (27.4)	0.274	4 (19.0)	7 (17.1)	9 (23.7)	8 (40.0)	0.253^*^
Hepatic dysfunction	28 (23.3)	11 (19.0)	17 (27.4)	0.274	7 (33.3)	8 (19.5)	10 (26.3)	3 (15.0)	0.509^*^
Portal hypertension	19 (15.8)	16 (27.6)	3 (4.8)	**0.001**	1 (4.8)	9 (22.0)	8 (21.1)	1 (5.0)	0.150^*^
Jaundice	17 (14.2)	8 (13.8)	9 (14.5)	0.910	0 (0.0)	2 (4.9)	6 (15.8)	9 (45.0)^ab^	**< 0.001** ^*^
**Neuropsychiatric**									
Tremor	78 (65.0)	36 (62.1)	42 (67.7)	0.515	11 (52.4)	27 (65.9)	24 (63.2)	16 (80.0)	0.318
Dystonia	70 (58.3)	35 (60.3)	35 (56.5)	0.666	9 (42.9)	24 (58.5)	25 (65.8)	12 (60.0)	0.398
Dysarthria	69 (57.5)	36 (62.1)	33 (53.2)	0.327	11 (52.4)	21 (51.2)	27 (71.1)	10 (50.0)	0.240
Gait abnormalities	63 (52.5)	30 (51.7)	33 (53.2)	0.869	10 (47.6)	19 (46.3)	26 (68.4)	8 (40.0)	0.115
Salivation	62 (51.7)	31 (53.4)	31 (50.0)	0.706	11 (52.4)	22 (53.7)	19 (50.0)	10 (50.0)	0.987
Ataxia	58 (48.3)	26 (44.8)	32 (51.6)	0.457	8 (38.1)	19 (46.3)	24 (63.2)	7 (35.0)	0.126
Memory impairment	55 (45.8)	21 (36.2)	34 (54.8)	**0.041**	4 (19.0)	20 (48.8)	20 (52.6)	11 (55.0)	0.055
Dysgraphia	54 (45.0)	25 (43.1)	29 (46.8)	0.686	10 (47.6)	16 (39.0)	20 (52.6)	8 (40.0)	0.627
Swallowing difficulty	46 (38.3)	23 (39.7)	23 (37.1)	0.773	9 (42.9)	12 (29.3)	19 (50.0)	6 (30.0)	0.223
Anxiety	44 (36.7)	20 (34.5)	24 (38.7)	0.631	4 (19.0)	17 (41.5)	15 (39.5)	8 (40.0)	0.329
Attention decrement	39 (32.5)	15 (25.9)	24 (38.7)	0.133	3 (14.3)	13 (31.7)	14 (36.8)	9 (45.0)	0.176
Sleep disorders	39 (32.5)	20 (34.5)	19 (30.6)	0.654	1 (4.8)	13 (31.7)	17 (44.7)^a^	8 (40.0)^a^	**0.015**
Postural abnormalities	39 (32.5)	22 (37.9)	17 (27.4)	0.219	9 (42.9)	13 (31.7)	13 (34.2)	4 (20.0)	0.473
Reduced facial expressiveness	39 (32.5)	16 (27.6)	23 (37.1)	0.266	8 (38.1)	12 (29.3)	17 (44.7)	2 (10.0)	0.053
Bradykinesia	33 (27.5)	15 (25.9)	18 (29.0)	0.698	7 (33.3)	8 (19.5)	14 (36.8)	4 (20.0)	0.273
Depression	28 (23.3)	12 (20.7)	16 (25.8)	0.508	4 (19.0)	11 (26.8)	9 (23.7)	4 (20.0)	0.916^*^
Personality changes	23 (19.2)	7 (12.1)	16 (25.8)	0.056	4 (19.0)	9 (22.0)	6 (15.8)	4 (20.0)	0.930^*^
Limb stiffness	18 (15.0)	7 (12.1)	11 (17.7)	0.384	6 (28.6)	4 (9.8)	6 (15.8)	2 (10.0)	0.253^*^
**Other systems**									
Joint pain	38 (31.7)	12 (20.7)	26 (41.9)	**0.012**	3 (14.3)	12 (29.3)	16 (42.1)	7 (35.0)	0.168

Data are presented as *n* (%) or median (*IQR*)

^a^ *P* < 0.05 when compared with ≤ 20 years of age at enrollment

^b^ *P* < 0.05 when compared with 21–30 years of age at enrollment

^c^ *P* < 0.05 when compared with 31–40 years of age at enrollment

^*^Fisher’s exact test

Bold values indicate statistically significant differences (*P* < 0.05)

### Mutation analysis of the ATP7B gene

The enrolled 120 WD patients were derived from 117 unrelated families. Among the 117 unrelated patients, 98 patients (83.8%) exhibited pathogenic mutations in both alleles, including 22 homozygotes and 76 compound heterozygotes. 17 patients (14.5%) had pathogenic mutations in one allele, while 2 patients (1.7%) were characterized with no pathogenic mutations. The overall mutation detection rate was 91.0%.

**Fig. 2:**
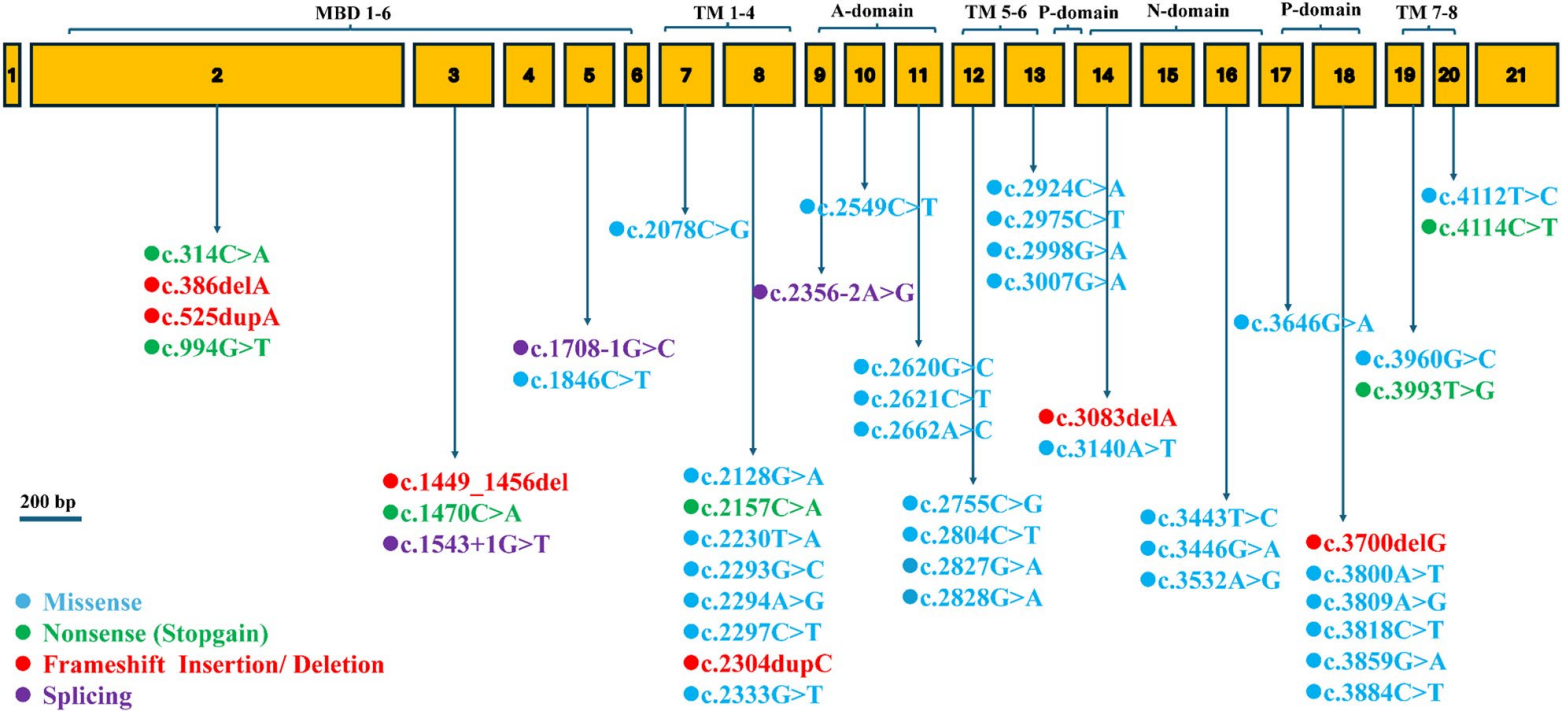
Spectrum of pathogenic ATP7B mutations in patients with WD from southwestern China

A total of 47 distinct pathogenic mutations were identified, including 32 missense mutations, 6 nonsense mutations, 6 frameshift mutations, and 3 splice-site mutations (Fig. 2). Notably, one novel pathogenic frameshift mutation, c.386delA (p.K129Rfs*24), was identified. Additionally, c.1987 C > A (p.P663T) is another novel mutation, which the InterVar tool (based on the ACMG/AMP 2015 guidelines) classified as a variant of uncertain significance.

As detailed in Supplementary Table S1, the c.2333G > T (p.R778L) mutation in exon 8 was the most prevalent, with an allelic frequency of 26.9% (63/234), followed by the c.2975 C > T (p.P992L) mutation in exon 13, with an allelic frequency of 16.2% (38/234). A total of 62 different pathogenic mutation combinations were identified (Supplementary Table S2), with the most common genotype being c.2333G > T/c.2333G > T (p.R778L/p.R778L), observed in 11.2% (11/98) of WD patients, followed by c.2333G > T/c.2975 C > T (p.R778L/p.P992L) in 9.2% (9/98). Additionally, 25 non-pathogenic mutations, which are unlikely to disrupt the normal function of the ATP7B gene or are classified as conflicting based on current evidence, were detected (Supplementary Table S3).

**Fig. 3:**
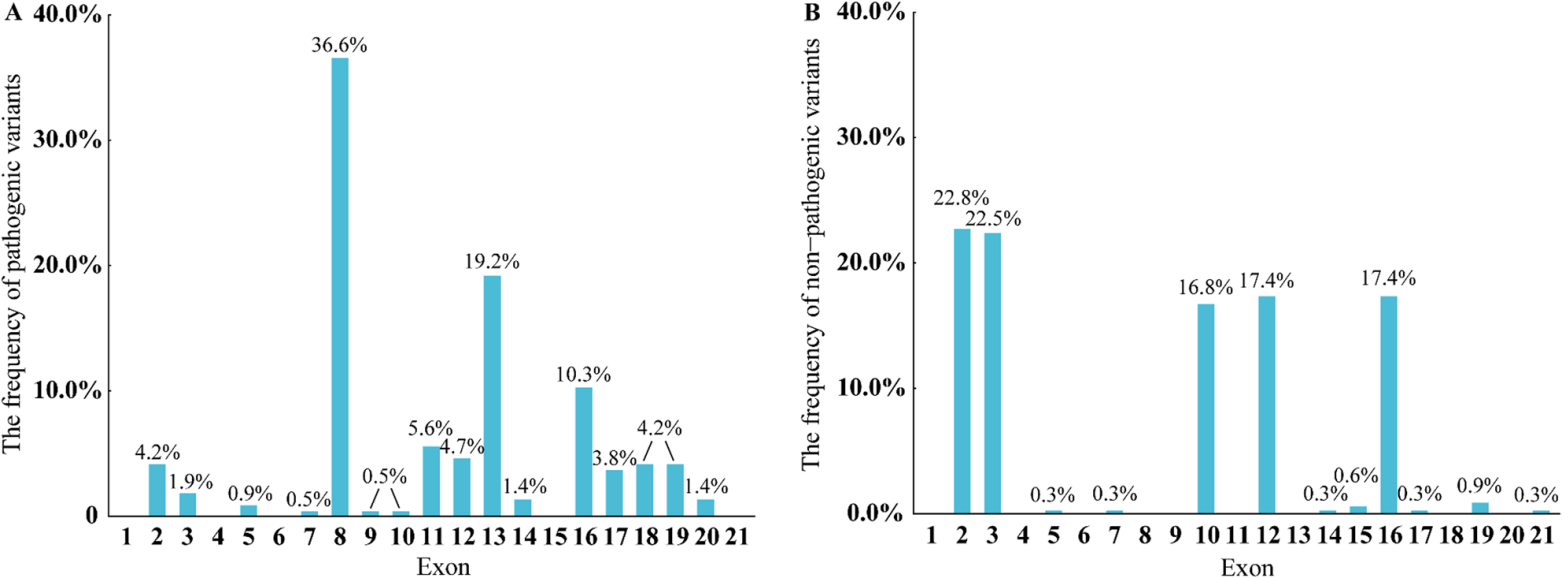
Distribution of mutations within 21 exons of ATP7B. **(A)** Pathogenic mutations, **(B)** Non-pathogenic mutations

These mutations were predominantly found in specific exons. Pathogenic mutations were most frequently detected in exon 8 (36.6%), followed by exon 13 (19.2%), exon 16 (10.3%), exon 11 (5.6%), and exon 12 (4.7%), which together accounted for 76.4% of all identified pathogenic mutations. Non-pathogenic mutations were most commonly found in exon 2 (22.8%), exon 3 (22.5%), exon 16 (17.4%), exon 12 (17.4%), and exon 10 (16.8%) (Fig. 3).

### Correlation between genotype and phenotype

**Fig. 4:**
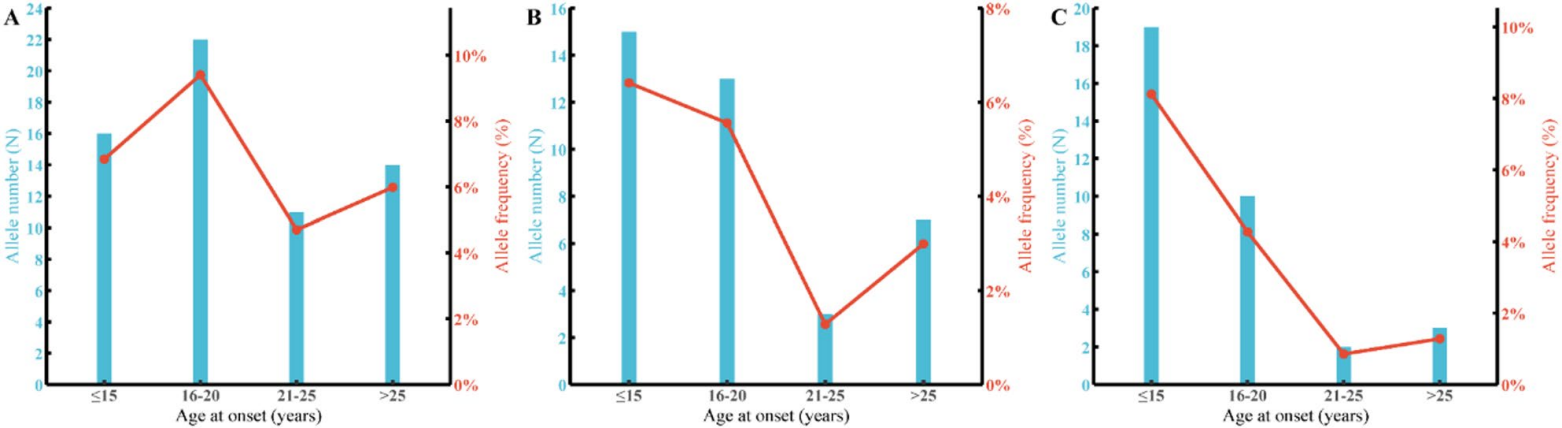
Allele frequency distribution by age at onset. (**A**) R778L, (**B**) P992L, and (**C**) LOF mutations

The distribution of R778L, P992L, and LOF mutations across different onset age groups was shown in Fig. 4. The age at onset in patients carrying the R778L and P992L mutations was distributed across all age groups, with the R778L allele showing the highest number and frequency in the 16–20 age group (Fig. 4A), whereas P992L exhibited the lowest frequency in the 21–25 age group (Fig. 4B). In contrast, LOF mutations were primarily detected in patients with an onset age of 20 years or younger and were rarely observed in those with onset after 20 years (Fig. 4C).

**Fig. 5:**
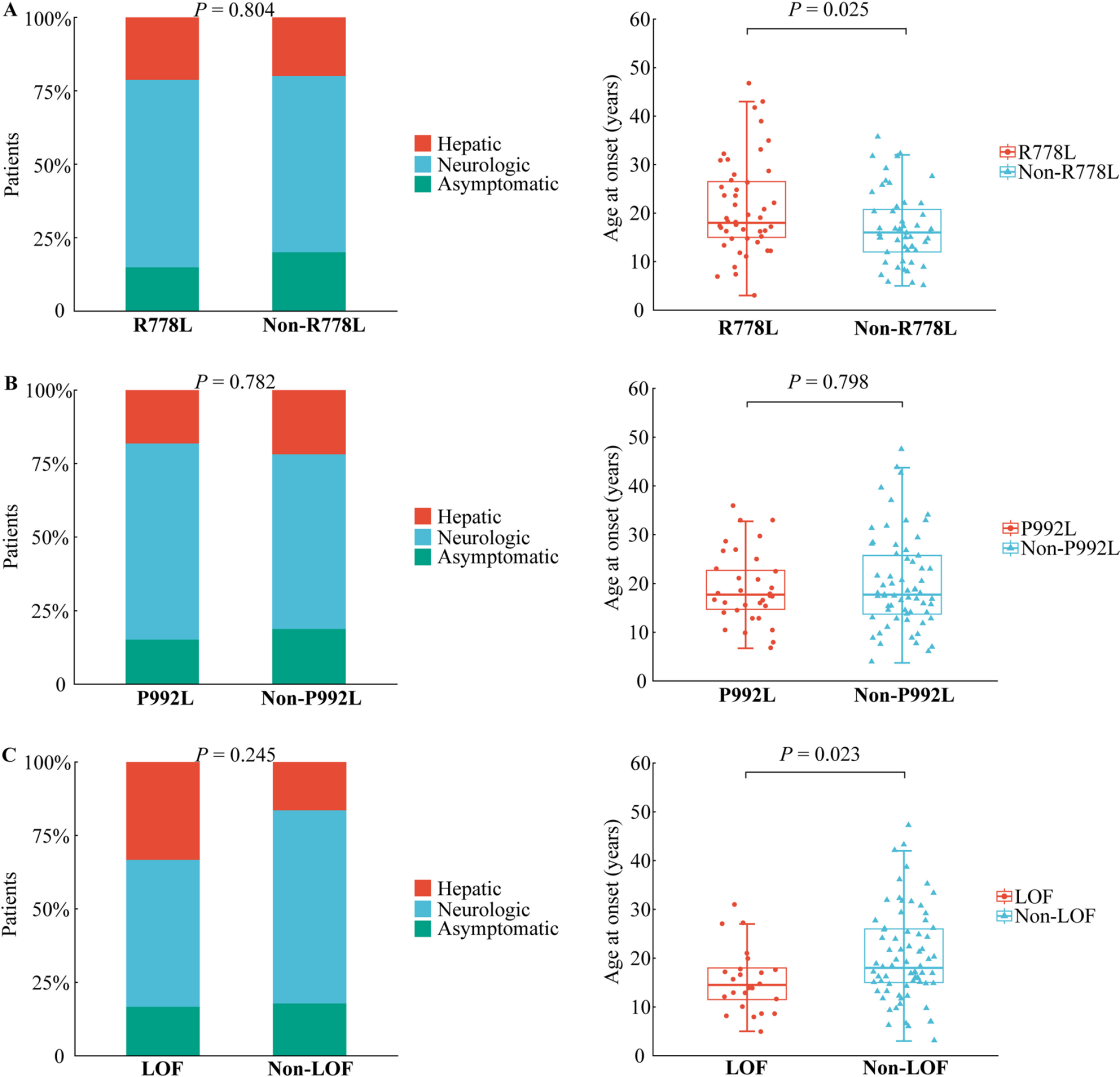
Comparison of initial clinical subtypes and age at onset among different genotypes. Left panels show the distribution of initial clinical subtypes, and right panels show the comparison of age at onset between carriers and non-carriers. (**A**) R778L, (**B**) P992L, and (**C**) LOF mutations

Among patients harboring pathogenic mutations on both alleles, comparisons of carrier status for R778L, P992L, and LOF mutations showed no significant differences in the distribution of initial clinical subtypes (all *P* > 0.05). In contrast, R778L carriers had a later age at onset than non-carriers (*P* = 0.025; Fig. 5A), whereas LOF carriers had an earlier age at onset (*P* = 0.023; Fig. 5C). No significantly association was observed between P992L carrier status and age at onset (*P* = 0.798; Fig. 5B).

The regression analyses were broadly consistent with these comparisons. In multinomial logistic regression models using asymptomatic onset as a reference, after adjusting for sex, carrier status for R778L, P992L, and LOF mutations was not significantly associated with either hepatic or neurologic onset (all *q* > 0.05; Table 3).

**Table 3:** Associations between genotype and initial clinical subtype

Mutation	Hepatic vs. Asymptomatic, *OR* (95% *CI*)	*P* value	*q* value	Neurologic vs. Asymptomatic, *OR* (95% *CI*)	*P* value	*q* value
R778L (ref: Non-R778L)	1.43 (0.39–5.27)	0.591	0.888	1.43 (0.48–4.25)	0.522	0.888
P992L (ref: Non-P992L)	1.04 (0.25–4.34)	0.953	0.969	1.39 (0.43–4.51)	0.581	0.888
LOF (ref: Non-LOF)	2.16 (0.51–9.17)	0.297	0.888	0.81 (0.22–2.97)	0.753	0.903

OR, odds ratio; CI, confidence interval; LOF, loss-of-function

In sex-adjusted linear regression models, R778L carrier status remained significantly associated with a later age at onset (*β* = 4.19, 95% *CI*: 0.66–7.73, *q* = 0.033), whereas LOF carrier status remained significantly associated with an earlier age at onset (*β* = -4.86, 95% *CI*: -8.99 to -0.74, *q* = 0.033). No significant association was identified between P992L carrier status and age at onset (Table 4). See Supplementary Table S4 for more details.

**Table 4:** Associations between genotype and age at onset

Mutation	*β* (95% *CI*)	*P* value	*q* value
R778L (ref: Non-R778L)	4.19 (0.66–7.73)	**0.021**	**0.033**
P992L (ref: Non-P992L)	-1.09 (-4.95–2.76)	0.574	0.574
LOF (ref: Non-LOF)	-4.86 (-8.99–-0.74)	**0.022**	**0.033**

CI, confidence interval; LOF, loss-of-function. Bold values indicate statistically significant differences (*P *< 0.05)

## Discussion

This study described the clinical characteristics and ATP7B gene mutations in patients with WD from southwestern China, and analyzed the correlation between genotype and phenotype.

Neurological manifestations were the most common initial presentation (61.7%), particularly tremor (41.7%), which is similar with findings from previous large Chinese cohorts [24, 27]. However, Ferenci P et al. reported hepatic disease as the more common initial presentation [32]. This difference may be attributed to differences in classification criteria. In our study, patients with neurological symptoms, regardless of the presence of liver symptoms, were classified as the neurologic subtype. Additionally, it could be related to factors such as region, ethnicity, sample selection, and healthcare resources. 20.0% of the patients were identified during physical examinations, primarily through liver function tests indicating elevation of transaminases, which is consistent with previous studies [23, 27]. Additionally, among the 120 patients, only one presented with other symptoms (nephritis) initially. Although other systemic symptoms are relatively rare, clinicians should still be aware of the multisystemic manifestations of WD to avoid misdiagnosis.

The median disease duration among the 120 patients was 10 years, and 62.5% of patients had progressed to cirrhosis. This progression is associated with worsening liver function and increased risk of complications, such as ascites (5.8%), esophagogastric varices (5.8%), hepatic encephalopathy (1.7%) and liver failure (3.3%) observed in this study. In a monocentric cohort from a French liver centre, 68% of patients were even already suffering from cirrhosis at diagnosis [33]. However, this finding should be interpreted cautiously, as our study was based on a single-center hospital cohort of patients receiving intravenous copper chelation therapy, which may have preferentially included patients with more severe or advanced disease. Neurological impairments were also commonly observed, including tremor (65.0%), dystonia (58.3%), and dysarthria (57.5%). Previous studies have shown that neurological symptoms in WD patients often persist over time, and while anti-copper treatment can slow disease progression, it cannot fully reverse existing neurological damage [34, 35]. Our findings were consistent with this. Among the 74 patients classified as the neurologic subtype at onset, 98.6% continued to experience neurological symptoms. Notably, neuropsychiatric symptoms later developed in 95.2% of patients with hepatic onset, compared with 66.7% of those initially identified incidentally during physical examination. Therefore, early detection and timely intervention are crucial for WD patients to preventing irreversible hepatic and neurological damage, as well as improving long-term outcomes.

We also observed differences in clinical manifestations according to sex and age. Female patients were more likely to experience fatigue, memory impairment, and joint pain, whereas portal hypertension was more prevalent in male patients. Since portal hypertension is a key feature of decompensated cirrhosis, this finding suggests that male patients may experience more severe liver damage compared to female patients. Similarly, previous studies have reported that the severity of nonalcoholic fatty liver disease (NAFLD) tends to be higher in males than in females, potentially due to the protective effects of estrogen [36]. Additionally, older patients showed higher frequencies of cirrhosis and jaundice. Among patients older than 40 years, the prevalence of cirrhosis even reached 90.0%, and jaundice occurred in 45.0%, suggesting that prolonged disease duration, cumulative copper toxicity, and declining hepatic compensatory function contribute to more severe liver impairment in older patients [37].

In addition to clinical characteristics, this study performed whole-genome resequencing on 120 patients from 117 unrelated families to analyze the characteristics of ATP7B gene mutations in WD patients from southwestern China. R778L and P992L were the most frequent ATP7B mutations, together accounting for 47.4% of all detected mutant alleles, which is consistent with previous reports in Chinese patients [22, 23, 38]. Pathogenic mutations with a frequency > 10% were mainly located in exons 8, 13, and 16, aligning with findings from southern China [23, 38]. However, pathogenic mutations in northern China are predominantly located in exons 8, 11, and 13 [22, 39], suggesting regional differences in mutation distribution. Non-pathogenic mutations were mainly located in exons 2, 3, 10, 12, and 16. Notably, exon 16 exhibited high frequencies of both pathogenic and non-pathogenic mutations, indicating that mutations in this exon should be interpreted cautiously regarding their clinical significance.

Two novel mutations were identified in this study: c.386delA (p.K129Rfs*24) and c.1987 C > A (p.P663T). We evaluated c.386delA according to the ACMG/AMP guidelines and the ClinGen Sequence Variant Interpretation recommendations for PVS1 [31]. This frameshift mutation is located in exon 2 of the reference ATP7B transcript (NM_000053.4/ENST00000242839.10). Under the PVS1 refinement framework, premature termination codons are generally predicted to escape NMD only when they occur in the last exon or within the 3′-most 50 nucleotides of the penultimate exon [31]. Therefore, the premature stop codon introduced by c.386delA is predicted to trigger NMD rather than escape it, which would be expected to result in degradation of the aberrant transcript and loss of function of the affected allele. Given that loss of ATP7B function is an established disease mechanism in WD, this mutation supports application of the PVS1 criterion [31, 40, 41]. In addition, because this mutation lies between MBD1 and MBD2, disruption of the normal organization of the N-terminal metal-binding region cannot be excluded. However, any specific effects on copper transport activity or interaction with Antioxidant 1 Copper Chaperone (Atox1) remain speculative in the absence of direct functional evidence [42, 43]. The c.1987 C > A (p.P663T) missense mutation is located in the Transmembrane Domain 1 (TM1) region. Previous studies of ATP7B have suggested that the integrity of the TM1/TM2 region is important for copper-responsive trafficking and exit from the TGN [44]. Therefore, p.P663T may affect local conformation, intracellular trafficking, or copper transport function. However, this remains a location-based hypothesis and requires functional validation. In this study, each of these two mutations was detected on a single allele in an individual patient, and their specific impact on ATP7B function requires further investigation.

Although pathogenic or likely pathogenic mutations were identified in most patients, genetic diagnosis was unresolved in some cases. 17 patients carried only one pathogenic/likely pathogenic mutation, and 2 patients exhibited no pathogenic/likely pathogenic mutations. This may be partly related to the stricter ACMG/AMP-based classification strategy used in the present study, under which mutations of uncertain significance were not counted as disease-causing. In addition, no exon-level copy number abnormalities of ATP7B were identified, although deep intronic or other non-coding mutations may still have contributed in some unresolved cases [45, 46]. As the present study focused primarily on exonic mutations, the potential splicing effects of intronic mutations were not systematically evaluated. Given the satisfactory alignment rate, sequencing depth, and genome coverage in our dataset, these unresolved cases are more likely attributable to the current analytical scope.

The relationship between ATP7B genotype and clinical phenotype in WD remains incompletely understood. In studies of Chinese cohorts, a higher age at onset has been associated with the neurological subtype, while no significant correlation was observed with sex, LOF mutations, or common missense mutations (R778L, P992L, and A874V) [27]. Another study reported an association between the R778L mutation and the hepatic subtype [47]. Regarding age at onset, patients harboring the P992L mutation tended to present symptoms earlier than those without this mutation on both alleles, whereas no significant association was found between R778L and age at onset [23]. However, a cohort study of 1,222 WD patients identified a correlation between R778L and younger age at onset [38]. This study similarly did not identify significant associations between carrier status for R778L, P992L, or LOF mutations and hepatic or neurological onset. However, R778L carrier status was associated with a later age at onset, whereas LOF carrier status was associated with an earlier age at onset. The discordant results across studies, particularly for R778L, may be attributable to differences in sample size, cohort composition, and genetic background, as well as to the complexity introduced by compound heterozygosity. Previous studies have suggested that different ATP7B mutations in compound heterozygotes may interact and influence each other’s functional effects [48]. Consistent with this possibility, a Chinese cohort reported that patients with the R778L/A874V genotype had a later onset than those with the R778L/R778L or R778L/P992L genotypes [49]. By comparison, LOF mutations are more likely than missense mutations to severely disrupt ATP7B function by preventing the production of a full-length protein. In this study, LOF mutations was associated with an earlier age at onset, in line with previous reports [32, 50, 51]. However, no correlation was observed between LOF mutations and clinical subtypes. This suggests that phenotypic variation may be influenced by additional genetic, epigenetic, or environmental factors that modulate the timing and presentation of disease symptoms [52, 53]. In conclusion, the ATP7B genotype alone cannot fully predict the age at onset, clinical manifestations, or disease progression of WD.

Our study has several limitations. First, this was a single-center hospital-based cohort, and all patients were recruited from those receiving intravenous copper chelation therapy, which may have enriched cases with more severe or advanced disease. This recruitment pattern may partly account for the high prevalence of cirrhosis and the predominance of neurologic presentations observed in our cohort. Second, the sample size of our study was small, which may introduce potential errors and influence the statistical outcomes. Third, although the study combined questionnaire interviews and medical record reviews to collect and verify clinical information, recall bias from patient self-reporting still existed. Finally, the genetic analysis focused on exonic mutations and did not systematically evaluate the potential splicing effects of intronic mutations, which may have contributed to unresolved molecular findings in some patients.

## Conclusion

This study described the clinical and genetic characteristics of WD patients in southwestern China. Tremor was the most common initial symptom, with most patients developing persistent neurological symptoms and cirrhosis. Two novel ATP7B mutations were identified, while R778L and P992L were the most common mutations. Regression analyses showed that R778L, P992L, and LOF mutations were not significantly associated with the initial clinical subtype, whereas R778L was associated with a later age at onset and LOF mutations with an earlier age at onset after adjustment for sex. These findings expand the ATP7B mutation spectrum with genetic data from southwestern China, and provide insights for early diagnosis and personalized management. However, multicenter and large-scale studies are needed to validate these conclusions.

## Supplementary Information

Below is the link to the electronic supplementary material.

Supplementary Material 1

Supplementary Material 2

Supplementary Material 3

Supplementary Material 4

## Data Availability

The data underlying this article will be shared on reasonable request to the corresponding author.

## Abbreviations

WD: Wilson’s disease
LOF: Loss-of-function
TGN: Trans-golgi networks
Cp: Ceruloplasmin
K-F: Kayser-fleischer
HGMD: The human gene mutation database
ACMG/AMP: The American college of medical genetics and genomics and the association for molecular pathology
NMD: Nonsense-mediated mRNA decay
FDR: False discovery rate
OR: Odds ratio
CI: Confidence interval
NAFLD: Nonalcoholic fatty liver disease
MBD: Metal-binding domain
Atox1: Antioxidant 1 copper chaperone
TM1: Transmembrane domain 1
