# Supplementary material for: Clinical characteristics and genetic features of patients with Wilson’s disease in southwestern China
Source: Orphanet J Rare Dis. 2026 Jun 3;21:274. doi: 10.1186/s13023-026-04426-y (PMC13455313; doi:10.1186/s13023-026-04426-y)
Supplement: Supplementary file 1 [file 13023_2026_4426_MOESM1_ESM.docx]

**Supplementary Table S1** Spectrum of pathogenic ATP7B mutations in patients with Wilson’s disease from southwestern China

| **Exon** | **cDNA change** | **Protein change** | **Mutation type** | **Protein domain*** | **AlphaMissense score** | **VARITY_R score** | **ACMG evidence** | **Class.** | **Hom.** | **Het.** | **Allele frequency (%)** |
| --- | --- | --- | --- | --- | --- | --- | --- | --- | --- | --- | --- |
| 2 | c.314C>A | p.S105X | Nonsense | MBD1 | - | - | PVS1, PM2, PP3, PP5 | P | 1 | 1 | 1.28 |
| 2 | c.386delA | p.K129Rfs*24 | Frameshift | MBD1/MBD2 | - | - | PVS1 | P | 0 | 1 | 0.43 |
| 2 | c.525dupA | p.V176Sfs*28 | Frameshift | MBD2 | - | - | PVS1 | P | 0 | 3 | 1.28 |
| 2 | c.994G>T | p.E332X | Nonsense | MBD3/MBD4 | - | - | PVS1, PM2, PP3, PP5 | P | 0 | 2 | 0.85 |
| 3 | c.1449_1456del | p.R483Sfs*20 | Frameshift | MBD4/MBD5 | - | - | PVS1 | P | 0 | 1 | 0.43 |
| 3 | c.1470C>A | p.C490X | Nonsense | MBD5 | - | - | PVS1, PM2, PP5 | P | 0 | 1 | 0.43 |
| 3 | c.1543+1G>T | - | Splicing | MBD5 | - | - | PVS1 | P | 0 | 2 | 0.85 |
| 5 | c.1708-1G>C | - | Splicing | MBD6 | - | - | PVS1 | P | 0 | 1 | 0.43 |
| 5 | c.1846C>T | p.R616W | Missense | MBD6 | 0.7719 | 0.926 | PS3, PM1, PM2, PP3, PP5 | P | 0 | 1 | 0.43 |
| 7 | c.2078C>G | p.S693C | Missense | TM2 | 0.7726 | 0.689 | PM1, PM2, PP3 | LP | 0 | 1 | 0.43 |
| 8 | c.2128G>A | p.G710S | Missense | TM2/TM3 | 0.5144 | 0.871 | PS3, PM1, PM2, PP3, PP5 | P | 1 | 0 | 0.85 |
| 8 | c.2157C>A | p.Y719X | Nonsense | TM2/TM3 | - | - | PVS1, PM2, PP3, PP5 | P | 0 | 1 | 0.43 |
| 8 | c.2230T>A | p.S744T | Missense | TM3 | 0.9328 | 0.546 | PM1, PM2, PM5, PP3 | LP | 0 | 2 | 0.85 |
| 8 | c.2293G>C | p.D765H | Missense | TM4 | 0.9722 | 0.935 | PM1, PM2, PM5, PP3 | LP | 0 | 1 | 0.43 |
| 8 | c.2294A>G | p.D765G | Missense | TM4 | 0.9684 | 0.883 | PM1, PM2, PP3, PP5 | LP | 0 | 1 | 0.43 |
| 8 | c.2297C>T | p.T766M | Missense | TM4 | 0.5767 | 0.861 | PM1, PM2, PM5, PP3 | LP | 0 | 1 | 0.43 |
| 8 | c.2304dupC | p.M769Hfs*26 | Frameshift | TM4 | - | - | PVS1 | P | 0 | 7 | 2.99 |
| 8 | c.2333G>T | p.R778L | Missense | TM4 | 0.9641 | 0.942 | PS3, PM1, PM2, PP3, PP5 | P | 11 | 41 | 26.92 |
| 9 | c.2356-2A>G | - | Splicing | TM4/A-domain | - | - | PVS1 | P | 0 | 1 | 0.43 |
| 10 | c.2549C>T | p.T850I | Missense | A-domain | 0.9033 | 0.862 | PM1, PM2, PP3, PP5 | LP | 0 | 1 | 0.43 |
| 11 | c.2620G>C | p.A874P | Missense | A-domain/TM5 | 0.9822 | 0.925 | PM1, PM2, PP3, PP5 | LP | 0 | 2 | 0.85 |
| 11 | c.2621C>T | p.A874V | Missense | A-domain/TM5 | 0.9281 | 0.651 | PS3, PM1, PM2, PP3 | LP | 0 | 5 | 2.14 |
| 11 | c.2662A>C | p.T888P | Missense | A-domain/TM5 | 0.832 | 0.984 | PM1, PM2, PP3, PP5 | LP | 0 | 5 | 2.14 |
| 12 | c.2755C>G | p.R919G | Missense | TM5 | 0.3244 | 0.721 | PS3, PM1, PM2, PP3, PP5 | P | 0 | 1 | 0.43 |
| 12 | c.2804C>T | p.T935M | Missense | TM5 | 0.6087 | 0.896 | PM1, PM2, PP3, PP5 | LP | 0 | 2 | 0.85 |
| 12 | c.2827G>A | p.G943S | Missense | TM5/TM6 | 0.7616 | 0.913 | PM1, PM2, PP3, PP5 | LP | 0 | 3 | 1.28 |
| 12 | c.2828G>A | p.G943D | Missense | TM5/TM6 | 0.993 | 0.913 | PM1, PM2, PP3, PP5 | LP | 1 | 2 | 1.71 |
| 13 | c.2924C>A | p.S975Y | Missense | TM6 | 0.983 | 0.984 | PM1, PM2, PP3, PP5 | LP | 0 | 1 | 0.43 |
| 13 | c.2975C>T | p.P992L | Missense | TM6 | 0.9934 | 0.982 | PS3, PM1, PM2, PP3, PP5 | P | 5 | 28 | 16.24 |
| 13 | c.2998G>A | p.G1000R | Missense | TM6 | 0.988 | 0.972 | PS3, PM1, PM2, PP3, PP5 | LP | 0 | 1 | 0.43 |
| 13 | c.3007G>A | p.A1003T | Missense | TM6/P-domain | 0.7895 | 0.882 | PM1, PM2, PP3, PP5 | LP | 0 | 1 | 0.43 |
| 14 | c.3083delA | p.K1028Rfs*93 | Frameshift | P-domain | - | - | PVS1 | P | 0 | 2 | 0.85 |
| 14 | c.3140A>T | p.D1047V | Missense | N-domain | 0.2059 | 0.604 | PM1, PM2, PP3, PP5 | LP | 0 | 1 | 0.43 |
| 16 | c.3443T>C | p.I1148T | Missense | N-domain | 0.5628 | 0.92 | PM1, PM2, PP3 | LP | 1 | 7 | 3.85 |
| 16 | c.3446G>A | p.G1149E | Missense | N-domain | 0.998 | 0.954 | PM1, PM2, PM5, PP3 | LP | 0 | 10 | 4.27 |
| 16 | c.3532A>G | p.T1178A | Missense | N-domain | 0.7214 | 0.712 | PM1, PM2, PP3, PP5 | LP | 0 | 3 | 1.28 |
| 17 | c.3646G>A | p.V1216M | Missense | P-domain | 0.4362 | 0.827 | PM1, PM2, PP3, PP5 | LP | 0 | 8 | 3.42 |
| 18 | c.3700delG | p.V1234Lfs*96 | Frameshift | P-domain | - | - | PVS1 | P | 0 | 1 | 0.43 |
| 18 | c.3800A>T | p.D1267V | Missense | P-domain | 0.9944 | 0.974 | PM1, PM2, PM5, PP3 | LP | 0 | 1 | 0.43 |
| 18 | c.3809A>G | p.N1270S | Missense | P-domain | 0.6947 | 0.921 | PS3, PM1, PM2, PP3 | LP | 0 | 3 | 1.28 |
| 18 | c.3818C>T | p.P1273L | Missense | P-domain | 0.5534 | 0.923 | PM1, PM2, PP3, PP5 | LP | 0 | 1 | 0.43 |
| 18 | c.3859G>A | p.G1287S | Missense | P-domain | 0.6613 | 0.888 | PM1, PM2, PP3, PP5 | LP | 0 | 1 | 0.43 |
| 18 | c.3884C>T | p.A1295V | Missense | P-domain | 0.9804 | 0.577 | PM1, PM2, PP3, PP5 | LP | 0 | 2 | 0.85 |
| 19 | c.3960G>C | p.R1320S | Missense | TM7 | 0.9154 | 0.894 | PM1, PM2, PP3, PP5 | LP | 0 | 2 | 0.85 |
| 19 | c.3993T>G | p.Y1331X | Nonsense | TM7 | - | - | PVS1, PM2 | LP | 2 | 3 | 2.99 |
| 20 | c.4112T>C | p.L1371P | Missense | TM8 | 0.9925 | 0.916 | PM1, PM2, PP3, PP5 | LP | 0 | 2 | 0.85 |
| 20 | c.4114C>T | p.Q1372X | Nonsense | TM8 | - | - | PVS1, PM2, PP3, PP5 | P | 0 | 1 | 0.43 |

*: The ‘/’ stands for located between structural domains. MBD, Metal binding domain; TM, Transmembrane domain; A-domain, Actuator domain; P-domain, Phosphorylation domain; N-domain, Nucleotide binding domain; Class., classification; P, pathogenic; LP, likely pathogenic; Hom., homozygous; Het., heterozygous. “-” indicates not applicable.
