# Supplementary material for: Clinical characteristics and genetic features of patients with Wilson’s disease in southwestern China
Source: Orphanet J Rare Dis. 2026 Jun 3;21:274. doi: 10.1186/s13023-026-04426-y (PMC13455313; doi:10.1186/s13023-026-04426-y)
Supplement: Supplementary file 2 [file 13023_2026_4426_MOESM2_ESM.docx]

**Supplementary Table S2**. ATP7B mutation combinations in this study.

| **Genotype** | ***n*** |  | **Genotype** | ***n*** |
| --- | --- | --- | --- | --- |
| c.2333G>T/c.2333G>T | 11 |  | c.2333G>T/c.2662A>C | 1 |
| c.2333G>T/c.2975C>T | 9 |  | c.2333G>T/c.3809A>G | 1 |
| c.2333G>T/c.3646G>A | 6 |  | c.2333G>T/c.3140A>T | 1 |
| c.2975C>T/c.2975C>T | 5 |  | c.2333G>T/c.3532A>G | 1 |
| c.2975C>T/c.3443T>C | 4 |  | c.2333G>T/c.1846C>T | 1 |
| c.2333G>T/c.2827G>A | 3 |  | c.2333G>T/c.2078C>G | 1 |
| c.2975C>T/c.3446G>A | 3 |  | c.2333G>T/c.2297C>T | 1 |
| c.2333G>T/c.2304dupC | 2 |  | c.2333G>T/c.2621C>T | 1 |
| c.3993T>G/c.3993T>G | 2 |  | c.2333G>T/c.2804C>T | 1 |
| c.1449_1456del/c.4114C>T | 1 |  | c.2333G>T/c.3884C>T | 1 |
| c.1543+1G>T/c.1708-1G>C | 1 |  | c.2333G>T/c.994G>T | 1 |
| c.2304dupC/c.3960G>C | 1 |  | c.2333G>T/c.3443T>C | 1 |
| c.2304dupC/c.3993T>G | 1 |  | c.2333G>T/c.3993T>G | 1 |
| c.2304dupC/c.4112T>C | 1 |  | c.2828G>A/c.2620G>C | 1 |
| c.3083delA/c.3800A>T | 1 |  | c.2828G>A/c.2828G>A | 1 |
| c.525dupA/c.3532A>G | 1 |  | c.3446G>A/c.3700delG | 1 |
| c.2662A>C/c.2157C>A | 1 |  | c.3446G>A/c.2621C>T | 1 |
| c.2662A>C/c.3859G>A | 1 |  | c.3446G>A/c.2620G>C | 1 |
| c.3532A>G/c.2356-2A>G | 1 |  | c.3646G>A/c.2621C>T | 1 |
| c.2975C>T/c.386delA | 1 |  | c.2230T>A/c.3884C>T | 1 |
| c.2975C>T/c.2662A>C | 1 |  | c.3443T>C/c.2304dupC | 1 |
| c.2975C>T/c.2549C>T | 1 |  | c.3443T>C/c.3443T>C | 1 |
| c.2975C>T/c.2621C>T | 1 |  | c.1470C>A/ c.3809A>G | 1 |
| c.2975C>T/c.2804C>T | 1 |  | c.2304dupC/c.3809A>G | 1 |
| c.2975C>T/c.2924C>A | 1 |  | c.2333G>T/Unknown | 5 |
| c.2975C>T/c.3818C>T | 1 |  | c.3446G>A/Unknown | 4 |
| c.2975C>T/c.2998G>A | 1 |  | c.525dupA/Unknown | 1 |
| c.2975C>T/c.3007G>A | 1 |  | c.2662A>C/Unknown | 1 |
| c.2975C>T/c.3960G>C | 1 |  | c.2621C>T/Unknown | 1 |
| c.2975C>T/c.994G>T | 1 |  | c.2828G>A/Unknown | 1 |
| c.2975C>T/c.2230T>A | 1 |  | c.3646G>A/Unknown | 1 |
| c.314C>A/c.1543+1G>T | 1 |  | c.3443T>C/Unknown | 1 |
| c.314C>A/c.314C>A | 1 |  | c.3993T>G/Unknown | 1 |
| c.2128G>A/c.2128G>A | 1 |  | c.4112T>C/Unknown | 1 |
| c.2293G>C/c.2755C>G | 1 |  |  |  |
| c.2333G>T/c.3083delA | 1 |  |  |  |
| c.2333G>T/c.525dupA | 1 |  |  |  |
| c.2333G>T/c.2294A>G | 1 |  |  |  |
