# Supplementary material for: Clinical characteristics and genetic features of patients with Wilson’s disease in southwestern China
Source: Orphanet J Rare Dis. 2026 Jun 3;21:274. doi: 10.1186/s13023-026-04426-y (PMC13455313; doi:10.1186/s13023-026-04426-y)
Supplement: Supplementary file 3 [file 13023_2026_4426_MOESM3_ESM.docx]

**Supplementary Table S3** Non-pathogenic ATP7B mutations in patients with Wilson’s disease from southwestern China

| **Location** | **cDNA change** | **Protein change** | **Mutation type** | **Hom.** | **Het.** | **Allele frequency (%)** |
| --- | --- | --- | --- | --- | --- | --- |
| UTR5 | c.-75C>A | - | - | 19 | 39 | 32.91 |
| Exon 2 | c.1168A>G | p.I390V | Missense | 0 | 1 | 0.43 |
| Exon 2 | c.1216T>G | p.S406A | Missense | 19 | 33 | 30.34 |
| Exon 3 | c.1366G>C | p.V456L | Missense | 19 | 33 | 30.34 |
| Exon 5 | c.1774A>T | p.I592F | Missense | 0 | 1 | 0.43 |
| Exon 7 | c.1987C>A | p.P663T | Missense | 0 | 1 | 0.43 |
| Exon 10 | c.2495A>G | p.K832R | Missense | 12 | 29 | 22.65 |
| Exon 12 | c.2785A>G | p.I929V | Missense | 0 | 1 | 0.43 |
| Exon 12 | c.2835C>T | p.I945I | Silent | 0 | 1 | 0.43 |
| Exon 12 | c.2855G>A | p.R952K | Missense | 12 | 29 | 22.65 |
| Exon 14 | c.3212T>C | p.L1071S | Missense | 0 | 1 | 0.43 |
| Exon 15 | c.3316G>A | p.V1106I | Missense | 0 | 1 | 0.43 |
| Exon 15 | c.3329A>C | p.E1110A | Missense | 0 | 1 | 0.43 |
| Exon 16 | c.3419T>C | p.V1140A | Missense | 12 | 29 | 22.65 |
| Exon 16 | c.3459G>T | p.W1153C | Missense | 0 | 1 | 0.43 |
| Exon 16 | c.3460C>T | p.L1154L | Silent | 0 | 1 | 0.43 |
| Exon 17 | c.3587A>G | p.D1196G | Missense | 0 | 1 | 0.43 |
| Exon 19 | c.3971A>G | p.N1324S | Missense | 0 | 1 | 0.43 |
| Exon 19 | c.3982G>A | p.A1328T | Missense | 0 | 2 | 0.85 |
| Exon 21 | c.4251A>G | p.T1417T | Silent | 0 | 1 | 0.43 |
| UTR3 | c.*1782C>G | - | - | 12 | 29 | 22.65 |
| UTR3 | c.*1578G>A | - | - | 0 | 1 | 0.43 |
| UTR3 | c.*1172G>A | - | - | 12 | 28 | 22.22 |
| UTR3 | c.*690C>G | - | - | 0 | 1 | 0.43 |
| UTR3 | c.*24G>A | - | - | 0 | 2 | 0.85 |

UTR, untranslated region; Hom., homozygous; Het., heterozygous. “-” indicates not applicable.
