# Supplementary material for: Clinical characteristics and genetic features of patients with Wilson’s disease in southwestern China
Source: Orphanet J Rare Dis. 2026 Jun 3;21:274. doi: 10.1186/s13023-026-04426-y (PMC13455313; doi:10.1186/s13023-026-04426-y)
Supplement: Supplementary file 4 [file 13023_2026_4426_MOESM4_ESM.docx]

**Supplementary Table S4** Associations between genotype and clinical phenotype

| **Phenotype** | **Comparison** | **Mutation** | **Crude estimate (95% *CI*)** | ***P* value** | ***q* value** | **Sex-adjusted estimate (95% *CI*)** | ***P* value** | ***q* value** |
| --- | --- | --- | --- | --- | --- | --- | --- | --- |
| Initial clinical subtype | Hepatic vs Asymptomatic | R778L (ref: Non-R778L) | 1.43 (0.39–5.26) | 0.592 | 0.888 | 1.43 (0.39–5.27) | 0.591 | 0.888 |
|  |  | P992L (ref: Non-P992L) | 1.03 (0.25–4.24) | 0.969 | 0.969 | 1.04 (0.25–4.34) | 0.953 | 0.969 |
|  |  | LOF (ref: Non-LOF) | 2.17 (0.52–9.09) | 0.291 | 0.888 | 2.16 (0.51–9.17) | 0.297 | 0.888 |
| Initial clinical subtype | Neurologic vs Asymptomatic | R778L (ref: Non-R778L) | 1.43 (0.48–4.25) | 0.521 | 0.888 | 1.43 (0.48–4.25) | 0.522 | 0.888 |
|  |  | P992L (ref: Non-P992L) | 1.39 (0.43–4.47) | 0.581 | 0.888 | 1.39 (0.43–4.51) | 0.581 | 0.888 |
|  |  | LOF (ref: Non-LOF) | 0.81 (0.22–2.94) | 0.752 | 0.903 | 0.81 (0.22–2.97) | 0.753 | 0.903 |
| Age at onset | – | R778L (ref: Non-R778L) | 4.19 (0.68–7.71) | **0.020** | **0.033** | 4.19 (0.66–7.73) | **0.021** | **0.033** |
|  |  | P992L (ref: Non-P992L) | -1.09 (-4.90–2.72) | 0.572 | 0.574 | -1.09 (-4.95–2.76) | 0.574 | 0.574 |
|  |  | LOF (ref: Non-LOF) | -4.77 (-8.85–-0.70) | **0.022** | **0.033** | -4.86 (-8.99–-0.74) | **0.022** | **0.033** |

For initial clinical subtype, estimates are odds ratios (ORs) derived from multinomial logistic regression with asymptomatic onset as the reference outcome category. For age at onset, estimates are regression coefficients (β values) derived from linear regression. Sex-adjusted models additionally included sex as a covariate. CI, confidence interval; LOF, loss-of-function. Bold values indicate statistically significant differences (*P*＜0.05).
